# The high temperature stress responses in the hepatopancreas of *Litopenaeus vannamei*: from immune dysfunction to metabolic remodeling cascade

**DOI:** 10.3389/fimmu.2025.1631655

**Published:** 2025-09-01

**Authors:** Yafei Duan, Meng Xiao, Yun Wang, Jianhua Huang, Yukai Yang, Hua Li

**Affiliations:** ^1^ State Key Laboratory of Mariculture Biobreeding and Sustainable Goods, Key Laboratory of South China Sea Fishery Resources Exploitation & Utilization, Ministry of Agriculture and Rural Affairs, South China Sea Fisheries Research Institute, Chinese Academy of Fishery Sciences, Guangzhou, China; ^2^ Key Laboratory of Efficient Utilization and Processing of Marine Fishery Resources of Hainan Province, Sanya Tropical Fisheries Research Institute, Sanya, China; ^3^ Shenzhen Base of South China Sea Fisheries Research Institute, Chinese Academy of Fishery Sciences, Shenzhen, China

**Keywords:** shrimp, high temperature, immunity, metabolites, gene expression

## Abstract

Sudden fluctuations in environmental temperatures are primarily caused by climate change. Aquatic organisms such as shrimp are poikilothermic animals, making them highly vulnerable to rising water temperatures, which can trigger stress responses and reduce aquaculture productivity. Hepatopancreas is of vital importance to the immunity, metabolism and detoxification of shrimp. In this study, the shrimp *Litopenaeus vannamei* were continuously exposed to high temperature (HT) stress at 33 °C for 7 days, and the hepatopancreatic histopathology, immune-related indexes, and metabolite patterns were explored. The results showed that HT stress caused abnormal morphological changes in the hepatopancreas of the shrimp, with the hepatic tubules becoming twisted, atrophied, and even ruptured and autolyzed. At the molecular level, stress-related indexes, such as *Nrf2*, *GPx*, and *HSP70* genes expression were increased, while *SOD* and *HSP90* genes were decreased; immune-related indexes, such as *ALF*, *Crus*, and *proPO* genes expression were increased, whereas *Pen3* gene was decreased; inflammation-related genes (*JNK* and *TNFα*) and apoptosis-related genes (*Casp9* and *Casp3*) expression were increased; autophagy-related indexes, such as *Atg3*, *Atg16*, and *Beclin1* genes expression were increased. Furthermore, HT stress caused the alterations in the metabolic patterns of the hepatopancreas, such as amino acid biosynthesis and metabolism, pentose and glucuronate interconversions, pantothenate and CoA biosynthesis, pyrimidine metabolism, and glycerophospholipid metabolism. Functional metabolites, such as tryptophan, arachidonic acid, cinnamic acid derivatives, vitamins, etc., were identified as biomarker candidates. The results revealed that HT stress induced comprehensive histomorphological and functional impairments in the hepatopancreas of *L. vannamei* through a cascade of oxidative damage, immune dysregulation, and metabolic disturbance.

## 1 Introduction

Pacific white shrimp *Litopenaeus vannamei* is one of the most widely cultivated shrimp species globally, which is critical for securing high-quality animal protein supplies and promoting the fishery economy (1). The global aquaculture production of *L. vannamei* exceeded 4.5 million tons in 2023, with China accounting for over 2.2 million tons. Shrimp are poikilothermic animals, highly susceptible to water temperature fluctuations. Both elevated and reduced temperatures can have significant impacts on shrimp, reducing their survival rate. Under the background of global warming, extreme high temperature (HT) events are becoming increasingly frequent, posing a huge challenge to the aquaculture industry (2, 3). In tropical and subtropical shrimp farming areas, the water temperature in summer usually exceeds 32 °C in shrimp ponds (4–6), sometimes reaching as high as 34 °C (7–9). HT stress can disrupt the physiological balance of shrimp as ectothermic animals, leading to metabolic disorders, weakened immunity, stunted growth, and even mass mortality, which seriously threatens shrimp farming and causes economic losses (10–14). Therefore, exploring the physiological responses of shrimp to HT stress is conducive to formulating anti-stress strategies.

HT stress has been shown to adversely impact shrimp, mainly focusing on inducing stress responses and disrupting immune homeostasis. For instance, HT stress can trigger oxidative stress responses in shrimp, leading to the accumulation of reactive oxygen species (ROS) and altering the activity of antioxidant enzymes (15, 16). HT stress can induce the up-regulation of heat shock protein (HSPs) gene expression in *L. vannamei*, including *HSP60*, *HSP70*, and *HSP90* (17, 18). Furthermore, HT stress can also disrupt the immune homeostasis of *L. vannamei* by affecting hemolymph osmolality, total hemocyte count (THC), and phenoloxidase activity (19). Additionally, HT stress compromises intestinal health of *L. vannamei* by damaging mucosal morphology, altering immune parameters, and inducing microbial community variations (1, 20).

HT stress also exerts notable impacts on shrimp metabolic processes. For instance, previous studies have demonstrated that HT stress influences glucose metabolism in the gills of *L. vanname*i, promoting a shift toward anaerobic carbohydrate utilization (21). Additionally, HT stress alters hemolymph glucose levels in *L. vannamei*, while having no significant impact on cholesterol, acylglycerol, or total protein contents (21). Metabolomic analyses further reveal that acute HT stress (33 °C) induces substantial metabolic alterations in the hemolymph of *L. vannamei*, particularly in the metabolism of “arachidonic acid”, “phenylalanine” and “alanine, aspartate and glutamate”, as well as in the biosynthesis of “phenylalanine, tyrosine and tryptophan” (1). Although numerous studies have explored the negative impacts of HT stress on shrimp health, the research remains insufficiently in-depth, lacking investigations into the mechanisms at different biological levels. A more comprehensive investigation is needed to elucidate the underlying mechanisms across multiple biological levels.

The hepatopancreas, as an important immune and metabolic organ of the shrimp, plays a key role in responding to environmental stress. In this study, we aim to systematically explore the effects of HT stress on the physiological functions in the hepatopancreas of *L. vannamei* by integrating immune indicators and metabonomics. Firstly, the morphological changes of the hepatopancreas were explored. Then, the characteristics of immune responses were analyzed based on the indicators related to stress, antibacterial, inflammation, apoptosis and autophagy. Finally, the metabolic pathways and potential metabolite markers were identified based on metabonomics methods. These results indicated that HT stress damaged the hepatopancreas structure and function of the shrimp via oxidative, immune, and metabolic disruption, which can provide insights for understanding HT stress adaptation and developing HT-resistant aquaculture strategies in shrimp.

## 2 Materials and methods

### 2.1 Shrimp and their rearing conditions

The shrimp *L. vannamei* used in this study were procured from an indoor shrimp pond in Shenzhen (China), with an average body weight of 6.3 ± 0.5 g. These shrimp had undergone strict pathogen detection, were specific pathogen-free, and had normal appearances without clinical symptoms of diseases. Before the HT stress exposure experiment, the shrimp individuals were acclimated for 7 days in tanks holding 300 L of aerated seawater. The rearing conditions were maintained through continuous water aeration and daily water exchange to ensure optimal water quality for shrimp culture, including a stable temperature of 28 ± 0.2 °C, pH 8.1-8.2, and salinity 30. The shrimp were fed commercial compound feed twice daily, with the feces and uneaten residues promptly removed from the tanks to maintain water cleanliness.

### 2.2 HT stress experiment and sampling

In this study, 33 °C was selected as the experimental temperature for HT stress exposure, based on actual shrimp farming practices in tropical and subtropical regions, as well as the previous research reports on high-temperature stress in shrimp (1, 20). After a 7-day acclimation period in tanks, the shrimp were randomly divided into two groups: a control (CK) group and a HT group. Each group consisted of three replicate tanks, with each tank containing 300 L of seawater and 50 shrimp. The CK group was maintained in normal seawater at a constant temperature of 28 ± 0.2 °C. For the HT group, the water temperature was set at 33 °C. A constant-temperature heater was used to gradually increase the temperature from 28 °C to 33 °C at a rate of 1 °C per hour, then maintained at constant temperature. Each tank’s water was changed daily. Prior to water exchange, we preheated the water to 33 °C in advance and then replaced the water in all the tanks of the HT group, thus preventing fluctuations in water temperature. All the rearing conditions except temperature were maintained consistently between the acclimation and experimental phases, with stable parameters of pH 8.1-8.2 and salinity 30. During the stress experiment, the shrimp were fed twice daily, with the feces and uneaten feed removed from the tank promptly.

After 7 days of stress, the hepatopancreas samples were collected randomly for analysis. Since shrimp are aquatic invertebrates with considerable individual variations, to reduce the differences among individuals, the hepatopancreas from five shrimp per tank were pooled and stored in RNAFollow solution for the mRNA expression analysis. For metabolomics analysis, two samples were collected from each tank, with each sample consisting of a mixture of the hepatopancreases from five shrimp, meaning there were six samples per group. Additionally, the hepatopancreas from three shrimp per tank was sampled for the histomorphological analysis.

### 2.3 Histomorphological analysis

Hepatopancreas samples were fixed in 4% paraformaldehyde for 24 h. After rinsing with running water for 30 min, the tissues were dehydrated through a series of ethanol solutions (70%, 80%, 90%, and 100%), washed with xylene, embedded in paraffin, and sectioned into 4 μm slices using a microtome (Leica RM2016, Shanghai). Following Hematoxylin and Eosin (H&E) staining, the sections were examined under a microscope (Nikon, Tokyo, Japan).

### 2.4 Gene expression analysis

Total RNA was isolated from the hepatopancreas samples using TRIzol reagent (Invitrogen, USA). Following the removal of genomic DNA and the purification of the RNA, cDNA synthesis was performed from the RNA using the Servicebio RT First-Strand cDNA Synthesis Kit (Wuhan, China). Real-time quantitative PCR (qPCR) was carried out with the SGExcel Fast SYBR qPCR Mix Kit (Sangon Biotech, China) on a Heal Force CG-02 qPCR system (Shanghai, China). The *β*-actin served as the reference gene, and the specific qPCR primer sequences are listed in **Supplementary Table S1**. Each sample underwent four technical replicates in the qPCR analysis. Relative mRNA expression levels of the target genes were calculated according to the method described by Livak and Schmittgen (22), presented as fold-changes relative to the CK group.

### 2.5 Non-targeted metabolomics analysis

Six hepatopancreas biological replicate samples per group were subjected to metabolomics analysis. After the pre-treatment of the hepatopancreas samples, the metabolites were extracted using a solution of methanol/chloroform and 2-chlorophenylalanine. Subsequently, the samples were detected by liquid chromatography-tandem mass spectrometry (LC-MS/MS). The liquid chromatography analysis was carried out on a Thermo Ultimate 3000 system, employing an ACQUITY UPLC HSS T3 (150 × 2.1 mm, 1.8 μm, Waters) chromatographic column. Mass spectrometry analysis was performed using a Thermo Q Exactive mass spectrometer. Data-dependent acquisition (DDA) MS/MS experiments were conducted using high-energy collision dissociation scans. To enhance data quality, dynamic exclusion was applied to filter out redundant information from the MS/MS spectra, ensuring the acquisition of highly relevant and accurate data.

Following the quality control, the metabonomic data were analyzed via partial least squares discriminant analysis (PLS-DA) to identify differential metabolites between the HT vs CK groups. Significance criteria were set as *P* < 0.05 and variable importance in projection (VIP) > 1.0. Agglomerative hierarchical clustering of the differential metabolites was performed using the pheatmap package in R software (v3.3.2). KEGG pathway annotation of differential metabolites was conducted using MetaboAnalyst software (www.metaboanalyst.ca), with subsequent analysis of metabolic pathways and interaction networks. Based on existing literature reports, we focused specifically on the differential metabolites with physiological and health-regulating functions, regarding them as potential biomarkers, and systematically analyzed their variation characteristics.

### 2.6 Statistical analysis

All the gene expression data were expressed as mean ± standard error (SE), and subjected to statistical analysis using one-way ANOVA with SPSS 27.0 software. A *P*-value < 0.05 was considered to denote statistical significance.

## 3 Results

### 3.1 Histomorphological changes of the hepatopancreas

**Figure 1:**
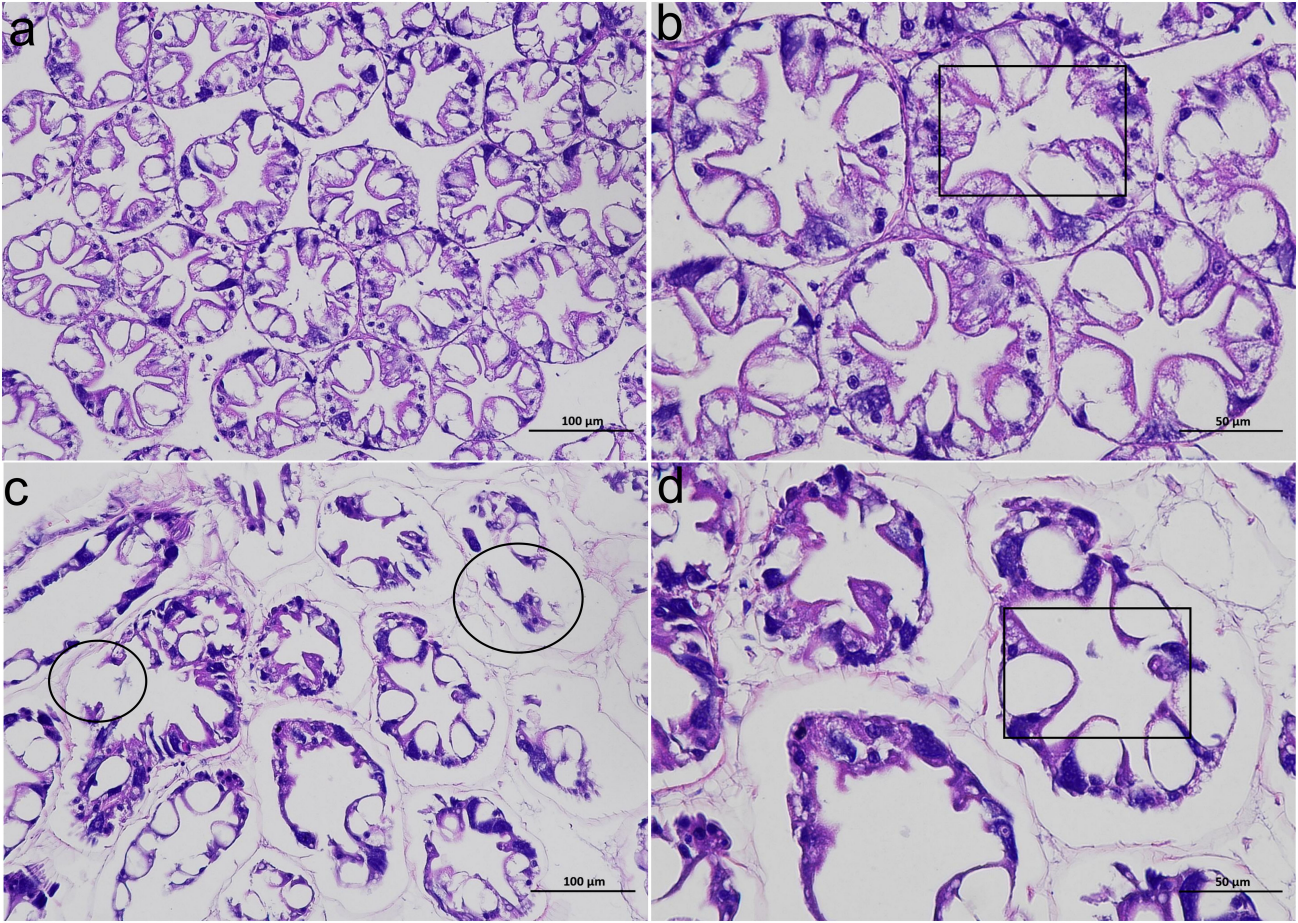
The alterations of the histological structure of the *L. vannamei* hepatopancreas after HT stress. **(a, b)** the CK group; **(c, d)** the HT group. **(a, c)** 200 ×; **(b, d)** 400 ×. A black box indicates the lumen; a black circle indicates a damaged hepatic tubule.

In the CK group, the hepatopancreatic tubules of the shrimp hepatopancreas showed relatively normal morphology, with tight connections and distinct star-shaped lumens (**Figures 1a, b**). However, in the HT group, the hepatopancreas exhibited abnormal morphological changes, such as irregular star-shaped structures of the hepatopancreatic tubules, which were twisted, atrophied, and detached from the basement membrane; some hepatopancreatic tubules even showed rupture and autolysis (**Figures 1c, d**). In the HT group, the diameter of hepatic tubules was significantly higher than that in the CK group (*P* < 0.05), while the lumen diameter showed a slight increase with no significant difference (*P* > 0.05) (**Supplementary Figure S1**), and the degeneration index reaching over 63%.

### 3.2 Changes in hepatopancreatic stress response indices

**Figure 2:**
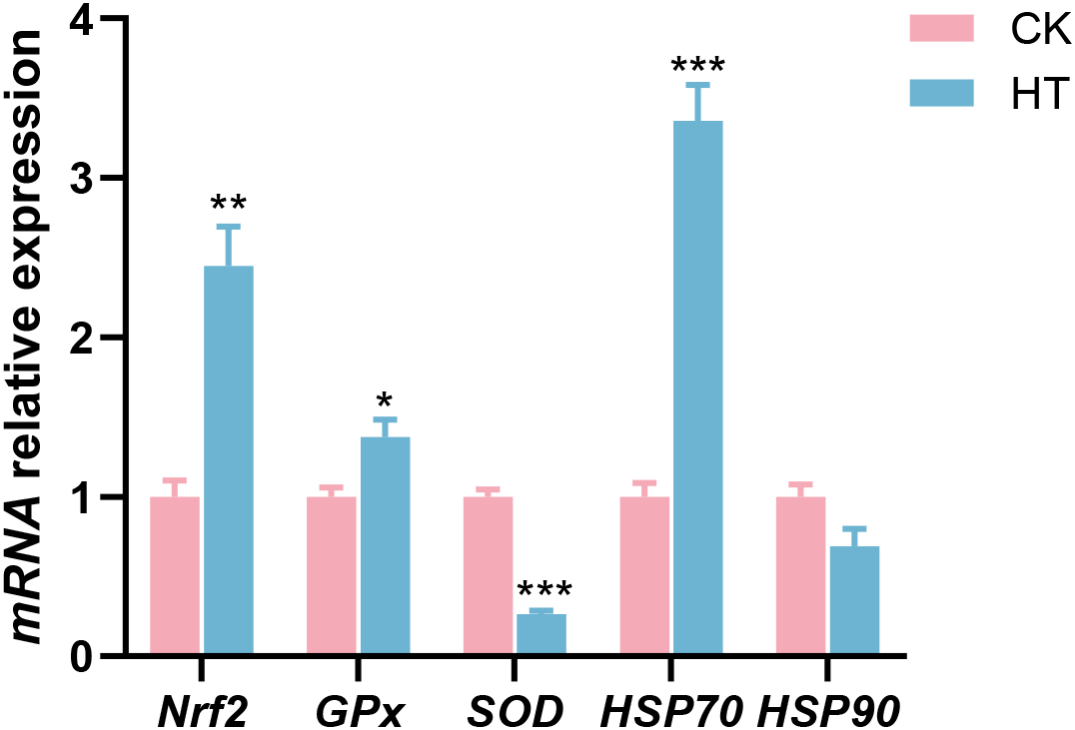
The alterations of the stress-related genes expression in the *L. vannamei* hepatopancreas after HT stress. The asterisk on the error bar show significant differences (*P < 0.05, **P < 0.01, ***P < 0.001).

Compared with the CK group, oxidative stress related indices, such as the relative mRNA expression levels of nuclear factor erythroid-derived 2-like 2 (*Nrf2*) and glutathione peroxidase (*GPx*) genes were significantly increased in the HT group (*P* < 0.05), while the expression of copper zinc superoxide dismutase (*SOD*) gene was significantly decreased (*P* < 0.05); stress related proteins, such as the relative mRNA expression levels of *HSP70* gene was significantly increased in the HT group (*P* < 0.05), while the expression of *HSP90* gene was slightly decreased with no statistical significance (*P* > 0.05) (**Figure 2**).

### 3.3 Changes in hepatopancreatic immunological indices

**Figure 3:**
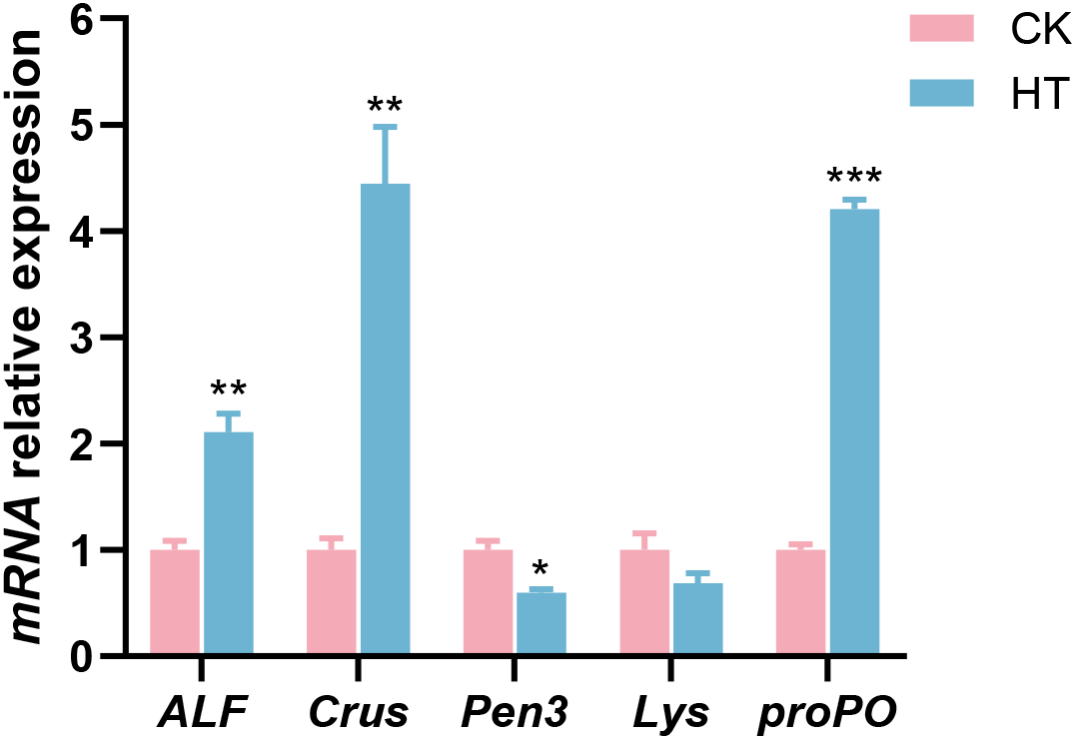
The alterations of the antibacterial-related genes expression in the *L. vannamei* hepatopancreas after HT stress. The asterisk on the error bar show significant differences (*P < 0.05, **P < 0.01, ***P < 0.001).

Compared with the CK group, immune related indices, such as the relative mRNA expression levels of anti-lipopolysaccharide factor AV-K (*ALF*), crustin (*Crus*), and prophenoloxidase (*proPO*) genes were significantly increased in the HT group (*P* < 0.05), while the expression of penaeidin 3a (*Pen3*) gene was significantly decreased (*P* < 0.05); the expression of lysozyme (*Lys*) gene was slightly decreased with no statistical significance (*P* > 0.05) (**Figure 3**).

### 3.4 Changes in hepatopancreatic inflammatory and apoptotic indices

**Figure 4:**
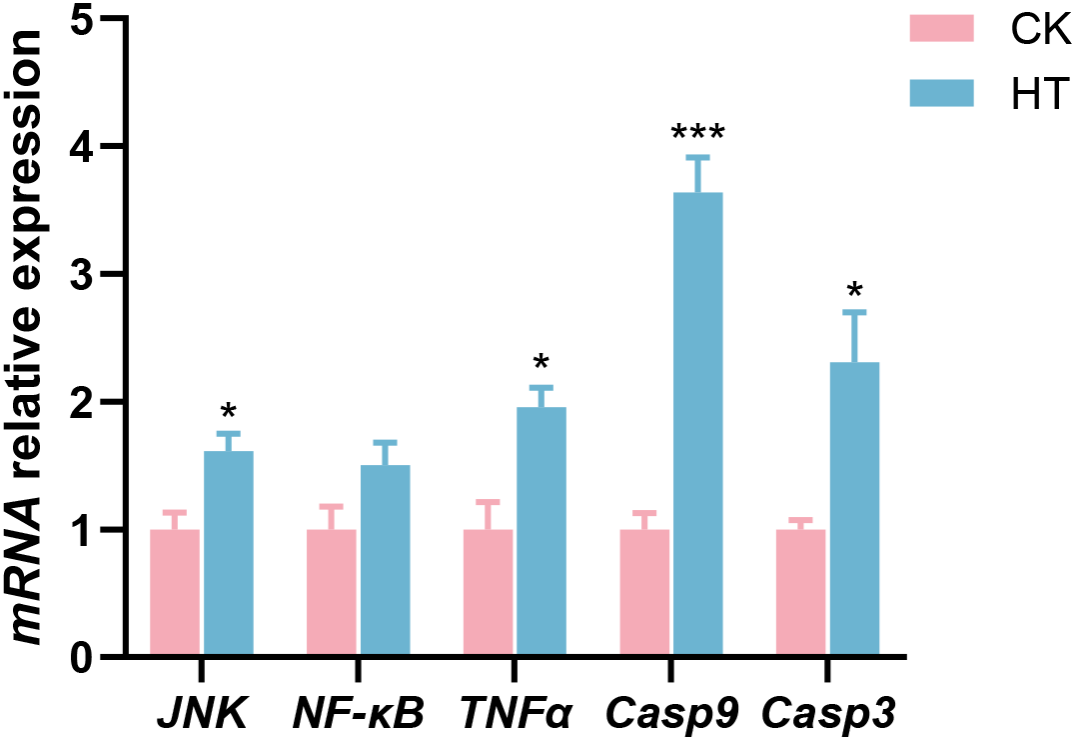
The alterations of the inflammation and apoptosis-related genes expression in the *L. vannamei* hepatopancreas after HT stress. The asterisk on the error bar show significant differences (*P < 0.05, ***P < 0.001).

Compared with the CK group, inflammatory related indices, such as the relative mRNA expression levels of c-Jun amino-terminal kinase (*JNK*) and tumor necrosis factor-α (*TNFα*) genes were significantly increased in the HT group (*P* < 0.05), while the expression of nuclear factor kappa-B (*NF-κB*) gene was slightly increased with no statistical significance (*P* > 0.05); apoptosis related indices, such as the relative mRNA expression levels of caspase-9 (*Casp9*) and caspase-3 (*Casp3*) genes were significantly increased (*P* < 0.05) in the HT group (**Figure 4**).

### 3.5 Changes in hepatopancreatic autophagic indices

**Figure 5:**
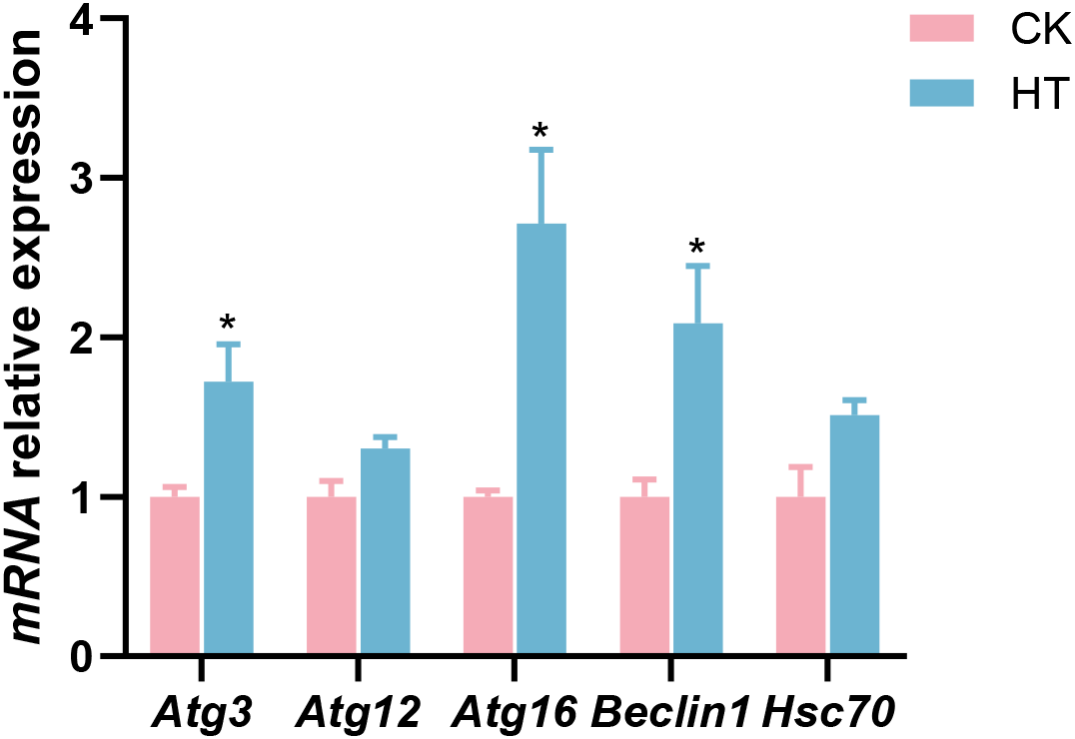
The alterations of the autophagy-related genes expression in the *L. vannamei* hepatopancreas after HT stress. The asterisk on the error bar show significant differences (*P < 0.05).

Compared with the CK group, autophagic related indices, such as the relative mRNA expression levels of autophagy-related protein 3 (*Atg3*), autophagy-related protein 16 (*Atg16*), and *Beclin1* genes were significantly increased in the HT group (*P* < 0.05), while the expression of autophagy-related protein 12 (*Atg12*) and heat shock cognate 70 (*Hsc70*) genes was slightly increased with no statistical significance (*P* > 0.05) (**Figure 5**).

### 3.6 Changes in hepatopancreatic metabolic patterns

#### 3.6.1 Functional analysis of differential metabolites

**Figure 6:**
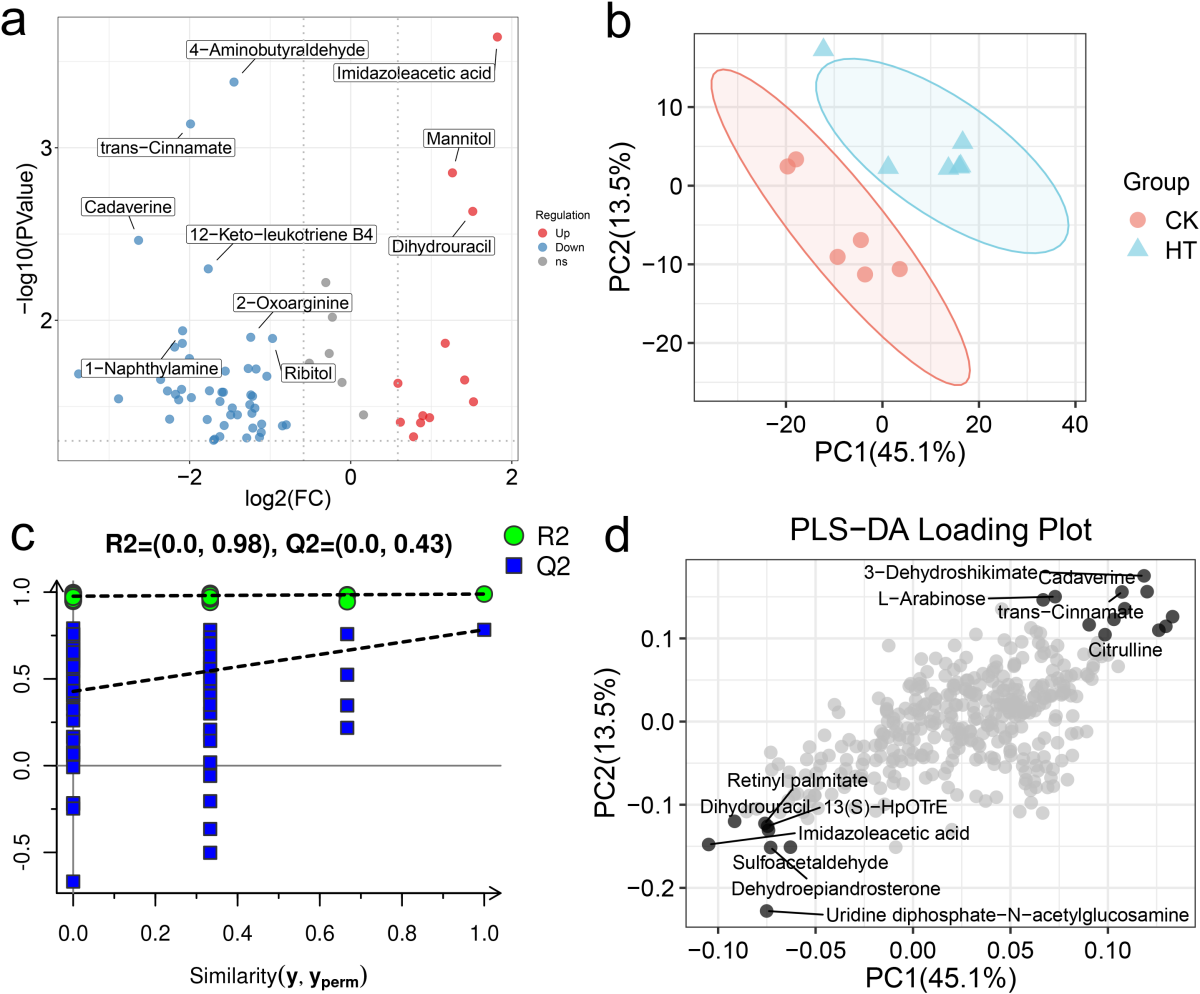
The alterations of the metabolite patterns in the *L. vannamei* hepatopancreas after HT stress. **(a)** the volcano plot of the metabolites; **(b)** the PLS-DA multivariate statistics of the metabolites; **(c)** the PLS-DA permutation test of the metabolites; **(d)** the PLS-DA loading plot of the metabolites.

The changes of metabolites in the hepatopancreas under HT stress were further analyzed (**Figure 6a**). Based on the multivariate statistical analysis of PLS-DA, there were obvious differences in the metabolic patterns between the HT and CK group (**Figures 6b–d**). Compared with the CK group, a total of 65 differential metabolites were identified in the HT group, including 52 up-regulated metabolites and 13 down-regulated metabolites (**Supplementary Figure S2**).

**Figure 7:**
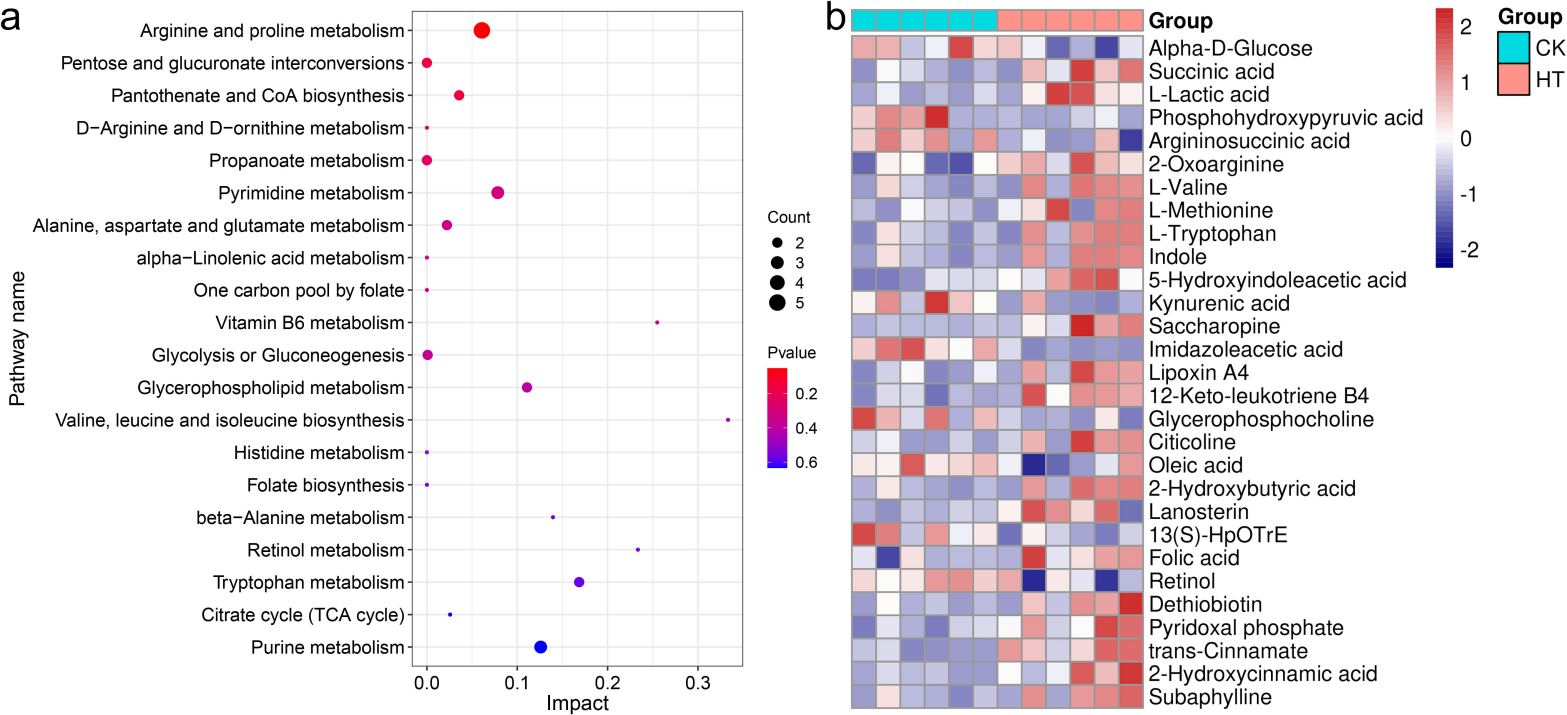
The enrichment pathways and candidate biomarkers of the differential metabolites in the *L. vannamei* hepatopancreas after HT stress. **(a)** the pathways of the HT vs CK group; **(b)** the candidate metabolite biomarkers.

The pathways involved in these differential metabolites were further analyzed. A total of 36 pathways were enriched, among them, “arginine and proline metabolism”, “valine, leucine and isoleucine biosynthesis”, “tryptophan metabolism”, “alanine, aspartate and glutamate metabolism”, “pentose and glucuronate interconversions”, “pantothenate and CoA biosynthesis”, “pyrimidine metabolism”, “glycerophospholipid metabolism” were highly enriched functions (**Figure 7a**).

**Figure 8:**
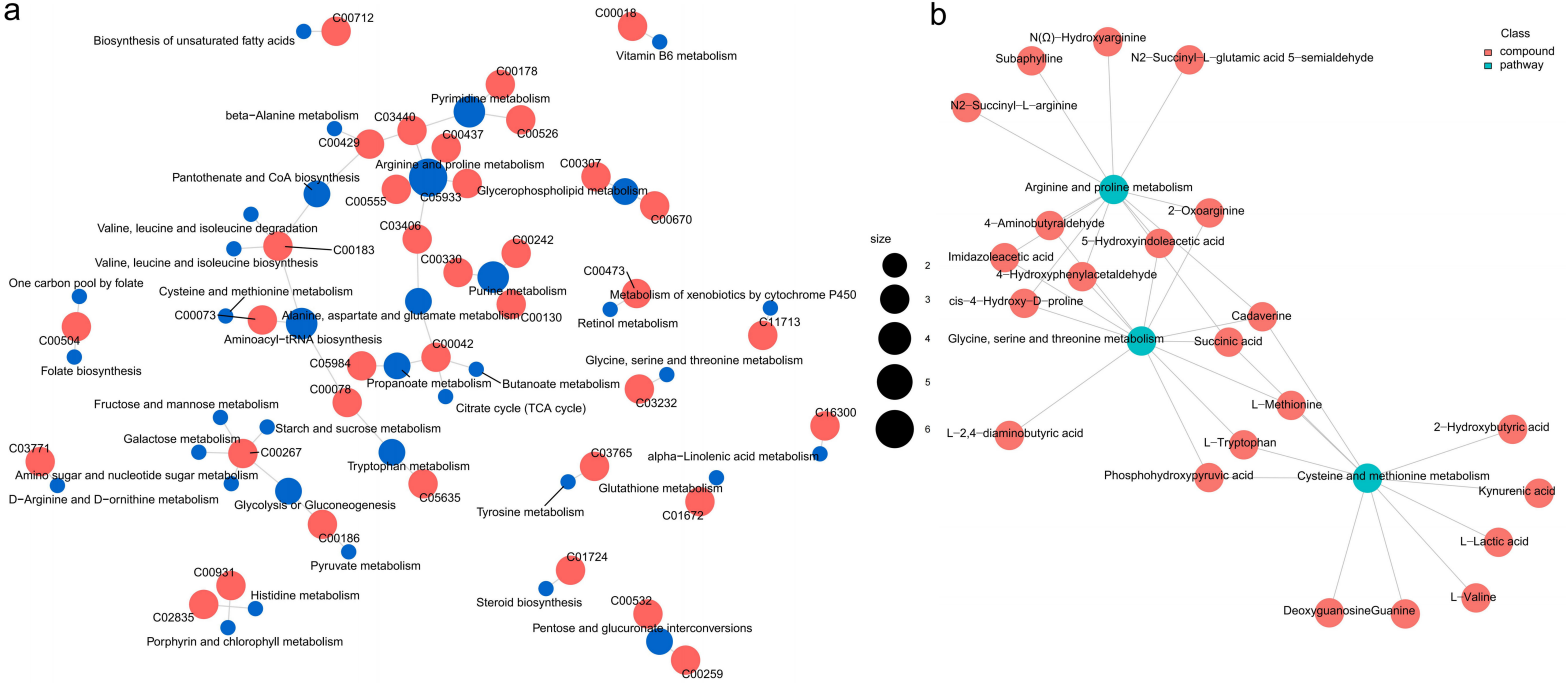
The relationship network between the differential metabolites and pathways in the *L. vannamei* hepatopancreas after HT stress. **(a)** the correlation network; **(b)** the FELLA enrichment network.

The network relationships among these highly enriched pathways were explored (**Figure 8a**). Of these, the pathway “arginine and proline metabolism” was correlated with “alanine, aspartate and glutamate metabolism” through the metabolite argininosuccinic acid (C03406); the pathway “aminoacyl-tRNA biosynthesis” was correlated with “tryptophan metabolism” and “cysteine and methionine metabolism” through the metabolites L-tryptophan (C00078) and L-methionine (C00073) respectively; the pathway “pyrimidine metabolism” was correlated with “pantothenate and CoA biosynthesis” through the metabolite dihydrouracil (C00429); the pathway “pantothenate and CoA biosynthesis”, “valine, leucine and isoleucine biosynthesis”, “alanine, aspartate and glutamate metabolism”, and “aminoacyl-tRNA biosynthesis” were correlated with each other through the metabolite L-valine (C00183). Furthermore, Based on the metabolomics-FELLA enrichment analysis, there was a high degree of correlation among the pathways “glycine, serine and threonine metabolism”, “arginine and proline metabolism”, and “cysteine and methionine metabolism” (**Figure 8b**).

#### 3.6.2 The change characteristics of differential metabolite markers

Several differential metabolite markers were rigorously screened (**Figure 7b**, **Table 1**). Among the three carbohydrate and derivatives, succinic acid and L-lactic acid were increased, but α-D-glucose was decreased. Among the eleven amino acid and derivatives, 2-oxoarginine, L-valine, L-methionine, L-tryptophan, indole, 5-hydroxyindoleacetic acid and saccharopine were increased, but phosphohydroxypyruvic acid, argininosuccinic acid, kynurenic acid and imidazoleacetic acid were decreased. Among the eight lipid and derivatives, lipoxin A4, 12-keto-leukotriene B4, citicoline, 2-hydroxybutyric acid, and lanosterin were increased, but glycerophosphocholine, oleic acid, and 13(S)-HpOTrE were decreased; of the four cofactors and vitamins, folic acid, dethiobiotin and pyridoxal phosphate were increased, but retinol was decreased. Among the three organic acids and derivatives, trans-cinnamate, 2-hydroxycinnamic acid, and subaphylline were increased.

**Table 1:** The potential metabolite biomarkers in the *L. vannamei* hepatopancreas after HT stress.

Metabolites	Log2 fold-change	VIP	Categories
α-D-glucose	-0.87	1.32	Carbohydrate and derivatives
Succinic acid	2.14	1.41	Carbohydrate and derivatives
L-lactic acid	2.17	1.42	Carbohydrate and derivatives
Phosphohydroxypyruvic acid	-1.52	1.28	Amino acid and derivatives
Argininosuccinic acid	-0.59	1.39	Amino acid and derivatives
2-oxoarginine	1.24	1.30	Amino acid and derivatives
L-valine	1.13	1.31	Amino acid and derivatives
L-methionine	1.22	1.66	Amino acid and derivatives
L-tryptophan	1.69	1.33	Amino acid and derivatives
Indole	0.85	1.44	Amino acid and derivatives
5-hydroxyindoleacetic acid	0.31	1.52	Amino acid and derivatives
Kynurenic acid	-0.98	1.42	Amino acid and derivatives
Saccharopine	3.38	1.38	Amino acid and derivatives
Imidazoleacetic acid	-1.82	1.92	Amino acid and derivatives
Lipoxin A4	1.47	1.39	Lipid and derivatives
12-keto-leukotriene B4	1.77	1.65	Lipid and derivatives
Glycerophosphocholine	-1.41	1.34	Lipid and derivatives
Citicoline	1.60	1.47	Lipid and derivatives
Oleic acid	-0.78	1.43	Lipid and derivatives
2-hydroxybutyric acid	1.49	1.43	Lipid and derivatives
Lanosterin	1.04	1.30	Lipid and derivatives
13(S)-HpOTrE	-1.17	1.51	Lipid and derivatives
Folic acid	0.80	1.35	Cofactors and Vitamins
Retinol	-0.62	1.59	Cofactors and Vitamins
Dethiobiotin	2.25	1.34	Cofactors and Vitamins
Pyridoxal phosphate	0.23	1.35	Cofactors and Vitamins
Trans-cinnamate	1.99	1.87	Organic acids and derivatives
2-hydroxycinnamic acid	2.19	1.50	Organic acids and derivatives
Subaphylline	1.78	1.33	Organic acids and derivatives

## 4 Discussion

During summer’s high-temperature seasons, the frequent stress problems in farmed shrimp have emerged as a critical constraint to aquaculture success. The physiological homeostasis of the shrimp organs is crucial for their defense against environmental stress, and the prerequisite for this is the integrity of histological morphology. Liao et al. reported that when *L. vannamei* is subjected to acute HT stress, the tissue morphology of the hepatopancreas exhibits significant damage (23). Such phenomena are also observed in this study, which will inevitably disrupt the physiological homeostasis of the shrimp hepatopancreas.

Oxidative stress serves as one of the key mechanisms contributing to the impacts of environmental stress on shrimp (24). As a key transcription factor, the Nrf2 regulates the gene expression of antioxidant enzymes (such as SOD and GPx) by binding to antioxidant response elements, thus playing a central regulatory role in safeguarding organisms against oxidative stress (25). HSP70 is a functional protein that can defend against oxidative stress (26). In this study, after HT stress, the expressions of *Nrf2*, *GPx*, and *HSP70* genes were increased in the hepatopancreas of the shrimp, while the expressions of *SOD* and *HSP90* genes were decreased. These findings indicated that HT stress triggered intracellular ROS accumulation, leading to oxidative stress in the hepatopancreas. The upregulation of the *Nrf2*/*GPx* signaling likely represented the organism’s primary defensive strategy against oxidative stress, while the downregulation of *SOD* might reflect stress-induced suppression of its expression. The *HSP70* and *HSP90* genes exhibited differential expression patterns, suggesting that the organism preferentially activated *HSP70* to mount a rapid stress response, while the downregulation of *HSP90* likely reflected resource reallocation under energy-limiting conditions.

Oxidative stress can induce autophagy via multiple signaling, which clears the organelles and proteins damaged by oxidative stress for intracellular stability (27). Autophagy-related genes (such as Atg3, Atg12 and Atg16) and Beclin1 play a crucial role in the autophagy process (28). Hsc70 drives chaperone-mediated autophagy by transporting substrate proteins to lysosomes for degradation (29). In this study, after HT stress, the expressions of *Atg3*, *Atg16* and *Beclin1* genes were significantly upregulated, and the expressions of *Atg12* and *Hsc70* genes also tended to be upregulated, indicating that the autophagy of hepatopancreatic cells in the shrimp was activated in response to the stress.

Environmental stress can affect the immune defense ability of aquatic animals. As an important component of the shrimp immune system, antimicrobial peptides can enhance the stress resistance of shrimp (30). The prophenoloxidase system participates in the melanization immune response in shrimp (31). In this study, HT stress induced the upregulation of *ALF* and *Crus* in the hepatopancreas of the shrimp, which could enhance immunity to cope with the damage caused by stress; the high expression of *proPO* was beneficial to tissue damage repair and the formation of a protective barrier. In contrast, the downregulation of *Pen3* and *Lys* reflected the selective allocation of immune resources, which might be due to the adaptive inhibition of their synthesis as energy was prioritized to ensure basic activities under HT stress. TNF-α, as an inflammatory mediator, can activate the JNK and NF-κB signalings, promote the expression of inflammation-related genes, and trigger an inflammatory response (32, 33). In this study, the upregulation of *JNK*, *NF-κB* and *TNFα* genes indicated that HT stress induced an inflammatory response in the hepatopancreas of the shrimp. The JNK signaling can activate the apoptotic factors Casp9 and Casp3 through various mechanisms, thereby inducing apoptosis (33, 34). In this study, the upregulation of *Casp9* and *Casp3* genes indicated that the apoptosis of the shrimp hepatopancreas was activated to cope with HT stress.

Metabolomics can quickly identify the physiological changes occurring in an organism by analyzing the alterations of metabolites. In this study, HT stress affected the metabolic function of the shrimp hepatopancreas, especially amino acid metabolism. Among them, changes in the metabolism of amino acids such as arginine, proline, and alanine might have been involved in immunity, osmotic regulation, and energy supply, while the metabolism of branched-chain amino acids and tryptophan might have supported protein repair and stress response. In addition, pentose conversion and glycerophospholipid metabolism affected carbohydrate utilization and membrane stability; adjustments in pantothenic acid and coenzyme A biosynthesis might have influenced stress resistance through energy metabolism. Similar phenomena also exist. For example, the amino acid metabolism in the hemolymph of the shrimp under heat stress at 33 °C for 72 h was also affected (1), but the specific types of amino acids affected were not completely the same as the results of our study, which might be related to different stress durations and tissue types.

Amino acids are crucial for maintaining the normal metabolism, physiological functions, and overall health of the organism. Tryptophan and its metabolites can regulate the immune response of aquatic animals and enhance their stress resistance (35). In this study, the increased of L-tryptophan, indole and 5-hydroxyindoleacetic acid, as well as the decreased of kynurenic acid, indicated that the tryptophan metabolism was involved in the response of the shrimp hepatopancreas to HT stress. Valine participates in protein synthesis, regulates blood sugar, supplies energy, and supports nervous system function (36). Methionine has the functions of synthesizing important biomolecules, detoxification and exerting antioxidant effects (37). In this study, the increased levels of L-valine and L-methionine might be the positive response of the shrimp hepatopancreas to HT stress, which was helpful to cope with the negative effects of the stress on the physiological homeostasis.

Lipids are essential for cellular energy supply, membrane integrity, and signaling (38). In this study, the glycerophospholipid metabolism pathway in the shrimp hepatopancreas was also disrupted. Glycerophospholipids are important lipid components of cell membranes (39). In this study, the decreased level of the glycerophosphocholine indicated that HT stress affected the homeostasis of the biological membranes of the hepatopancreas cells by influencing lipid homeostasis. Arachidonic acid and its metabolites are important immunoregulatory substances (40). In this study, the increased levels of lipoxin A4 and 12-keto-leukotriene B4 implyed that HT stress might also affect the immune homeostasis of the shrimp hepatopancreas through the metabolites of arachidonic acid.

Organic acids have physiological functions such as regulating energy metabolism and enhancing immunity. Cinnamic acid exhibits effects of regulating immunity, antioxidant activity, and anti-inflammation (41); subaphylline is a derivative of hydroxycinnamic acid (42). In this study, the increased levels of trans-cinnamate, 2-hydroxycinnamic acid, and subaphylline might contribute to enhancing the ability of the shrimp hepatopancreas to defend against high-temperature stress. 2-hydroxybutyric acid is involved in energy metabolism and immune regulation, and improves drug-induced liver injury (43). In this study, the elevated level of 2-hydroxybutyric acid might be beneficial for coping with the hepatopancreatic injury in the shrimp caused by HT stress. Fluctuations in vitamin levels were also closely related to the metabolic function of the hepatopancreas. In this study, after HT stress, the increased levels of folic acid, dethiobiotin, and pyridoxal phosphate might have been involved in key processes such as one-carbon unit transfer and amino acid metabolism under stress conditions, providing coenzyme support for cell repair and the synthesis of immune molecules. In contrast, the decreased level of retinol, a precursor of vitamin A might have been related to the increased demand for epithelial cell repair that it is involved in, and the accelerated consumption might have indirectly reflected the degree of damage to the hepatopancreas tissue.

## 5 Conclusion

**Figure 9:**
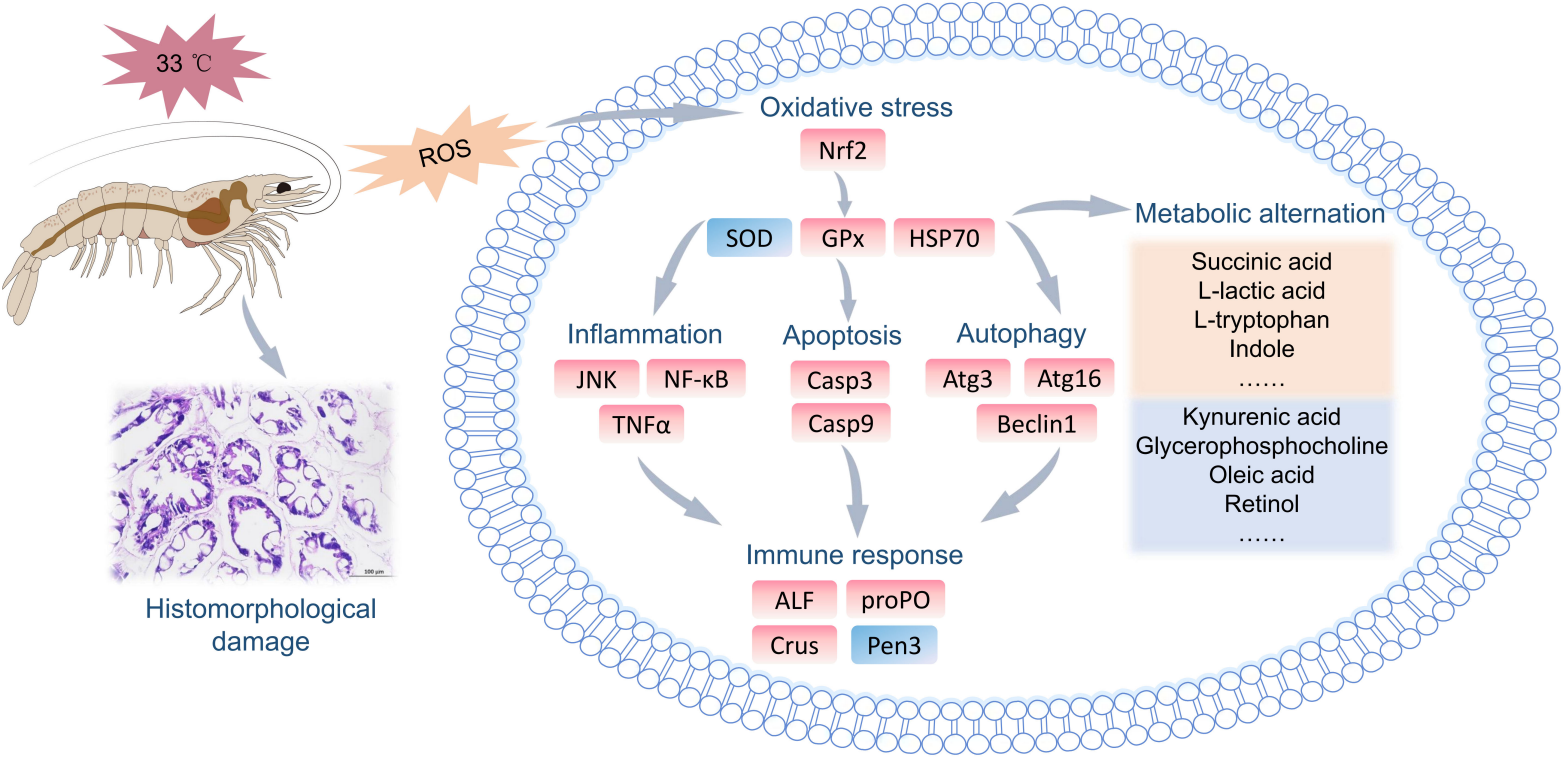
A mechanistic deduction of the effects of HT stress on the functional homeostasis of the hepatopancreas in *L. vannamei*.

This study revealed the negative effects of 33 °C HT stress on the hepatopancreas of *L. vannamei*. HT stress induced the structural damage to the hepatopancreatic tubules, such as distortion, atrophy, and even rupture. At the molecular level, HT stress activated stress responses (*Nrf2*, *GPx*, *HSP70*), and the resulting oxidative stress further induced the upregulation of genes related to inflammation (*JNK*, *TNFα*), apoptosis (*Casp3*, *Casp9*) and autophagy (*Atg3*, *Atg16*, *Beclin1*), thereby causing the disordered expression of immune genes. Additionally, metabolomic profiling indicated the disturbances in crucial metabolic pathways including amino acid metabolism, pentose interconversion, and glycerophospholipid metabolism, with tryptophan and arachidonic acid identified as potential biomarkers. We inferred that HT stress induced oxidative stress, caused immune dysregulation, activated inflammatory and cell death as well as autophagy pathways, led to hepatopancreatic metabolic disorders. This eventually triggered hepatopancreatic structural damage and the impairment of functional homeostasis (**Figure 9**).

## Data Availability

The original contributions presented in the study are included in the article/**Supplementary Material**. Further inquiries can be directed to the corresponding author.
