# Supplementary material for: The high temperature stress responses in the hepatopancreas of Litopenaeus vannamei: from immune dysfunction to metabolic remodeling cascade
Source: Front Immunol. 2025 Sep 1;16:1631655. doi: 10.3389/fimmu.2025.1631655 (PMC12433876; doi:10.3389/fimmu.2025.1631655)
Supplement: Supplementary file 1 [file DataSheet1.doc]

**Supplementary materials**

**Table S1** Primer sequences used in this study.

| Gene | Forward primer (5’-3’) | Reverse primer (5’-3’) | Source |
| --- | --- | --- | --- |
| *Nrf2* | GATGAGAAGCGAGCCAGAGCG | GCCGTCGGATGTCTCGGATAA | Fish and Shellfish Immunology 133 (2023) 108547 |
| *SOD* | GACACGACCATTAGCCTGTACGAC | CAGCGTTGCCAGTAGCGAGTG | HM371157.1 |
| *GPx* | TCTGAGCGGCGAGATGGTGTC | CTGGTGGAAGTCCCTGGTGGTC | AY973252.2 |
| *HSP70* | TGTGCCTGCCTACTTCAACGATTC | CGCTCACCGCCAACCTTCTTG | AY645906.1 |
| *HSP90* | TTCGGCGTGGGCTTCTACTCC | TACTGCTCGTCGTCGTTGTTCTTG | HQ008268.1 |
| *ALF* | GGTGTTCCTGGTGGCACTCT | AGCTCCGTCTCCTCGTTCCT | GQ227486.1 |
| *Crus* | TGGTGTAGGTGGCGGTCTTGG | CTTGTGGGCAGTCGAGTATCTTGG | AY486426.1 |
| *Pen3* | CACCACCCTTCGTGAGACCTTTG | GTGACAGCAACGCCCTAACCG | AF390139.1 |
| *Lys* | TCGAGTCGTCCTTCAACACG | TGCAGACGTTCTTGCCGTAG | AY170126.2 |
| *proPO* | CAATGACCAGCAGCGTCTTC | CACGGAAGGAGGCGTATCAT | AY723296.1 |
| *JNK* | TCTACCTGGTGATGGAGCTGATGG | CGGCTGAGTGTAAGTGCTTTATCCC | JN035903.1 |
| *NF-κB* | TCTAACCAATCACCACAGCAC | TGGTAAACTCAGTGTTCGGG | Fish and Shellfish Immunology 133 (2023) 108547 |
| *TNFα* | CTCAGCCATCTCCTTCTTG | TGTTCTCCTCGTTCTTCAC | Aquaculture 559 (2022) 738370 |
| *Casp9* | ATGGCTCGTGGTTCATTCAG | CATCAGGGTTGAGACAATACAGG | Science of the Total Environment 879 (2023) 163039 |
| *Casp3* | AGACGGACAGCATACAGGAGGAC | CTCGGCCAAGAAGTGGATGAAGAC | EU421939.1 |
| *Atg3* | TGGAGTATCGCAGTGAGCAGGAG | TGCCATGTCAGCCACTTTCTCTTC | MH797018.1 |
| *Atg12* | CGTAAACAATGGAGGGCGAGAAGG | TTCTCCTGCTCCTGGTTTTCTTGTG | XM_027363883.1 |
| *Atg16* | ACAGTAAAGGAGGTGGAGGAGATGG | CCGTCTGTGGAGGTCCGTTAATTC | XM_027371533.1 |
| *Beclin1* | GCGACCTACACACAGTATGCCTAAC | CCATCCTCCATGCTCTCCAACAAG | XM_027382390.1 |
| *Hsc70* | GTCGCCTTCACAGACACAGA | GAATTTGCGGCCGATCAGTC | EF495128.1 |
| *β-actin* | TCGCTCCCTCCACCATGAAGATC | CTCCTGCTTGCTGATCCACATCTG | AF300705.2 |

**
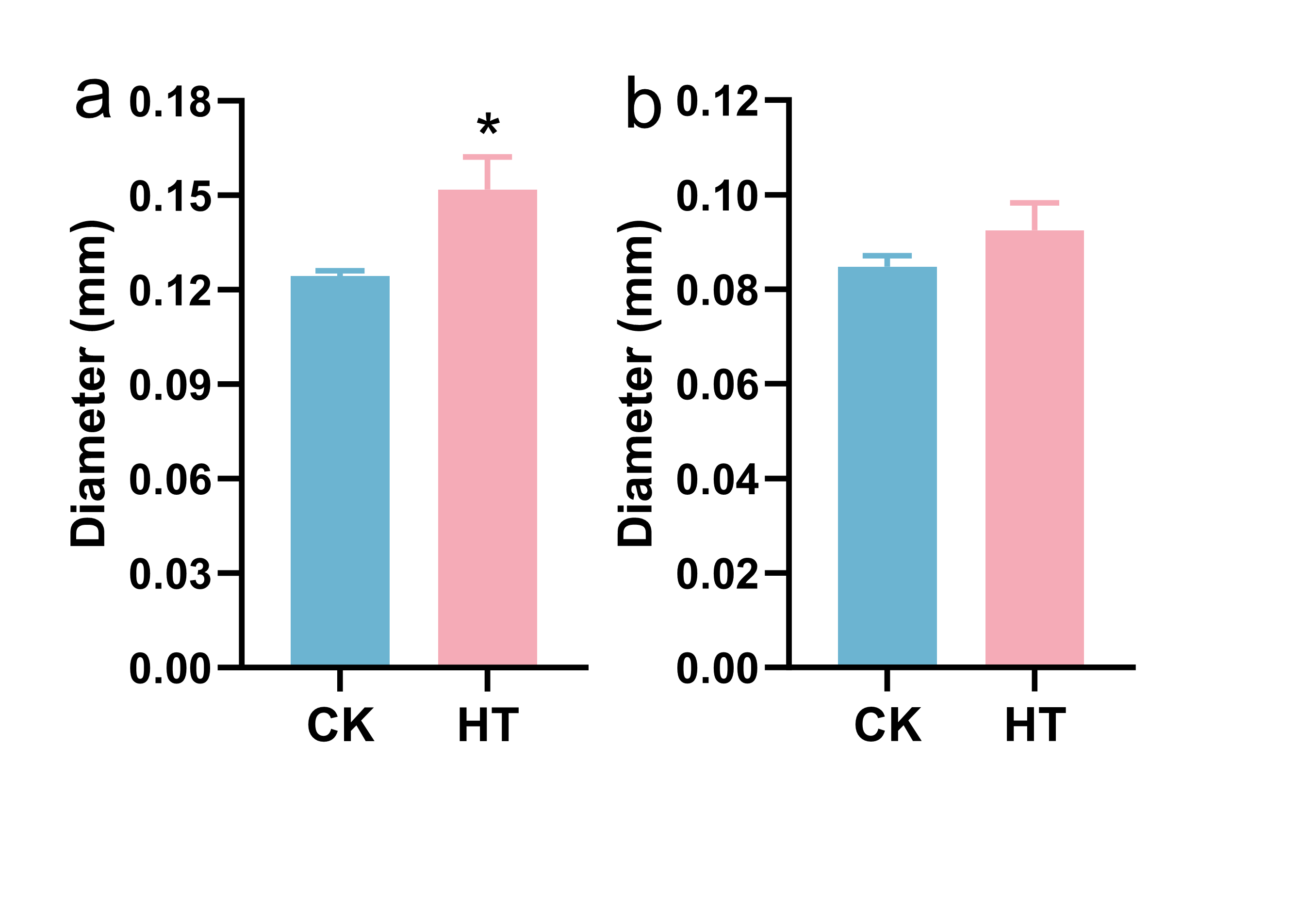
**

**Figure S1** The changes of the diameter of hepatopancreatic tubules and lumens in *L. vannamei* after HT stress. (a) the diameter of hepatopancreatic tubules; (b) the diameter of lumens. The asterisk on the error bar show significant differences (**P* < 0.05).

**
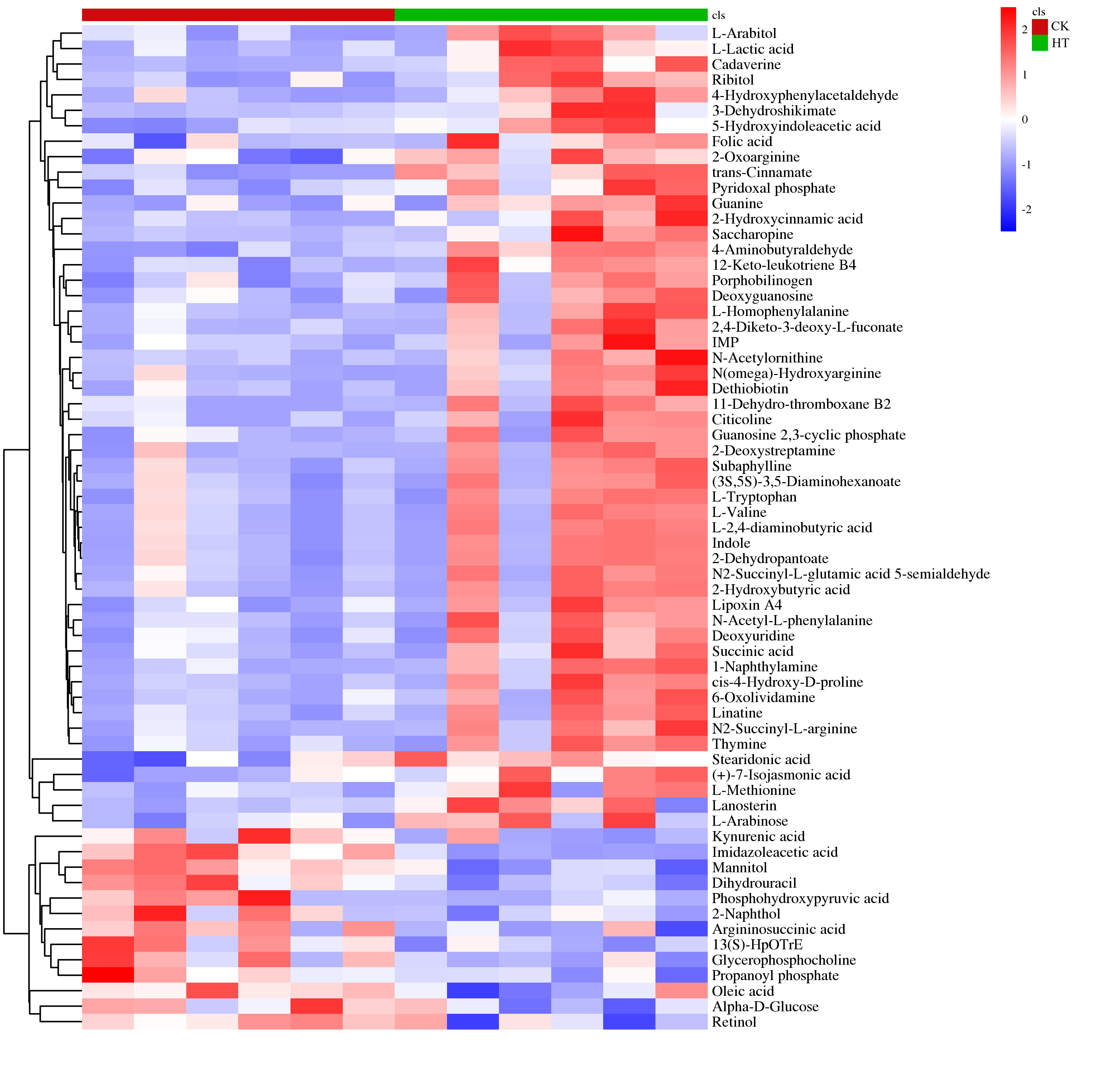
**

**Figure S2** The clustering of differential metabolites in the *L. vannamei* hepatopancreas after HT stress*.*
